# Proteomic features characterization of *Hymenoptera* venom allergy

**DOI:** 10.1186/s13223-019-0387-5

**Published:** 2019-11-27

**Authors:** Eliza Matuszewska, Joanna Matysiak, Anna Bręborowicz, Katarzyna Olejniczak, Zdzisława Kycler, Zenon J. Kokot, Jan Matysiak

**Affiliations:** 1 Department of Inorganic and Analytical Chemistry, Poznan University of Medical Sciences, 6 Grunwaldzka Street, 60-780 Poznan, Poland; 2 Medical Faculty, Higher Vocational State School, 13 Kaszubska Street, 62-800 Kalisz, Poland; 3 Department of Pulmonology, Pediatric Allergy and Clinical Immunology, Poznan University of Medical Sciences, 27/33 Szpitalna Street, 60-572 Poznan, Poland

**Keywords:** *Hymenoptera* venom, MALDI, Protein-peptide profiling, Allergic inflammatory response, Sting

## Abstract

**Background:**

*Hymenoptera* venom allergy is one of the most frequent causes of anaphylaxis. In its most severe form, the reaction to wasp and honey bee stings may be life-threatening. Therefore, immediate and proper diagnosis of venom allergy and implementation of suitable therapy are extremely important. Broadening the knowledge on the mechanism of the allergic reaction may contribute to the improvement of both diagnostic and treatment methods. Thus, this study aimed to discover changes in protein expression in serum of patients allergic to *Hymenoptera* (wasp and honeybee) venom and to point out proteins and peptides involved in the allergic inflammation.

**Methods:**

Serum proteomic patterns typical to allergic patients and healthy volunteers were obtained with MALDI-TOF (matrix-assisted laser desorption/ionization-time of flight) mass spectrometer. The spectra were processed, analyzed and compared using advanced bioinformatics tools. The discriminative peaks were subjected to identification with liquid chromatography coupled with tandem mass spectrometry.

**Results:**

This methodology allowed for the identification of four features differentiating between allergy and control groups. They were: fibrinogen alpha chain, coagulation factor XIII chain A, complement C4-A, and inter-alpha-trypsin inhibitor heavy chain H4. All of these proteins are involved in allergic inflammatory response.

**Conclusions:**

Extending the knowledge of the *Hymenoptera* venom sensitization will contribute to the development of novel, sensitive and specific methods for quick and unambiguous allergy diagnosis. Understanding the basis of the allergy at the proteomic level will support the improvement of preventive and therapeutic measures.
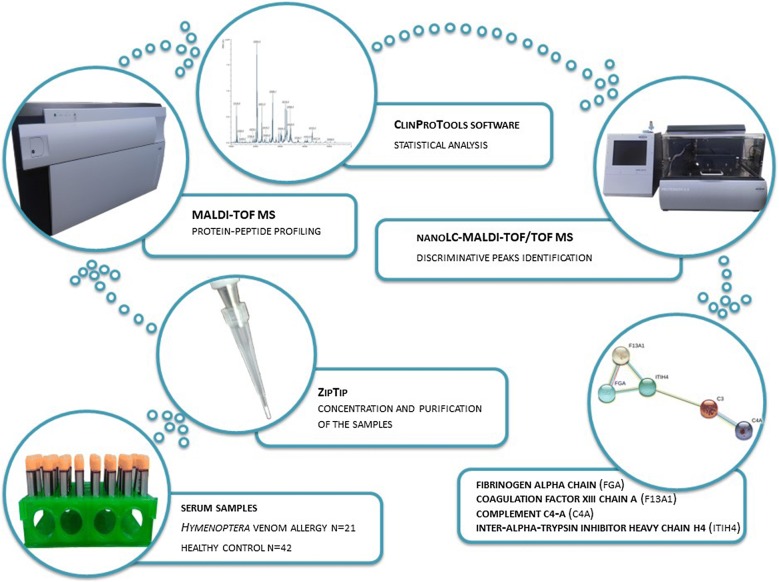

## Background

*Hymenoptera* venom allergy, along with drug and food allergic reactions, is one of the most frequent causes of anaphylaxis worldwide. Wasp (*Vespula vulgaris, Vespula germanica*) and honeybee (*Apis mellifera*) stings are very frequent and manifest in a variety of clinical symptoms, but the anaphylactic shock is the most dramatic and occasionally fatal reaction [1, 2]. The risk of anaphylaxis significantly reduces the quality of life. Therefore the immediate and proper diagnosis of *Hymenoptera* venom allergy and application of suitable therapy are extremely important.

The first step in *Hymenoptera* venom allergy diagnosis is a detailed patient interview and medical examination which allows classifying the reaction: allergic or non-allergic, local or systemic. Patients with systemic reaction are classified for complementary diagnostic tests, as subsequent sting may cause even more serious consequences [3, 4]. Based on the clinical symptoms, results of diagnostic tests and quality of life, patients are qualified or disqualified for venom immunotherapy (VIT). However, widely applied diagnostic methods, such as venom specific immunoglobulin E (IgE) tests and skin tests (both skin prick tests and intracutaneous tests), may not correlate with clinical symptoms and cannot be enough for qualification to VIT [5]. Therefore, it is important to understand the mechanism and molecular consequences of allergy to the venom. Detailed knowledge of clinical and immune mechanisms in *Hymenoptera* venom allergy may lead to better diagnosis and application of appropriate treatment [6].

The study of the molecular mechanism of the diseases and the development of effective diagnostic methods require advanced analytical strategies. One of the most innovatory approaches aiming in complete understanding of the processes occurring in the living organisms is proteomics. Discovery-based proteomics is concerned with protein-peptide profiling and identification of distinctive proteomic patterns. By the application of modern mass spectrometry techniques, this approach enables to assess how protein composition change in time regarding environmental and genetic conditions. Thus, the compilation of proteomic data explains the molecular basis of pathogenesis [7].

This research aimed to discover changes in protein expression in patients allergic to *Hymenoptera* venom and to point out proteins and peptides involved in allergic inflammation. It was reported, that serum profiles change after the *Hymenoptera* insect sting, in both humans and animals (i.e. rats) [8, 9]. Analysis of protein-peptide profiles typical to allergic patients and healthy volunteers were performed using MALDI-TOF (matrix-assisted laser desorption/ionization-time of flight) mass spectrometer. Despite the increasing importance of proteomic profiling as a strategy of assessing the clinical significance of proteins involved in disease processes, in the available literature there are no reports considering *Hymenoptera* venom allergy. This study is the first attempt to compare protein/peptide patterns characteristic to allergic patients and healthy subjects with an eye to pointing out proteins responsible for an allergic response.

## Methods

### Study groups and serum samples

The participants of the study were 21 patients diagnosed with an allergy to *Hymenoptera* (wasp and honey bee) venom—test group and 42 healthy volunteers—control group. After explanation of the assumption of the study and the possible consequences, written informed consent was obtained from all the subjects, and in case of children, from their parents. The project was approved by the Bioethical Commission of Poznan University of Medical Sciences (decision No. 324/11).

Demographic profiles of participants are shown in Table 1. All participants fulfilled a detailed survey and underwent a medical examination. Based on clinical symptoms and venom specific IgE (sIgE) levels, the individuals were divided into the study groups. Allergic patients—had clinical symptoms after the sting: large local reactions or/and systemic symptoms and have positive diagnostic tests: venom specific IgE and/or skin tests. Control group—never have been stung in the past or had local reactions after the sting (normal reaction or large local reaction) and have negative diagnostic tests—venom specific IgE—class 0. In both allergic patients and controls, sIgE levels were determined by ImmunoCap (Phadia AB, Uppsala, Sweden). Specific IgE were estimated to wasp venom and honeybee venom, sIgE to cross-reactive carbohydrate determinant (CCD) were estimated to MUXF3 (neo-glycoprotein fucosylated/xylosylated *N*-glycans) from bromelain. Moreover, sIgE were determined to the species-specific recombinant major allergens (rSSMA): phospholipase A1 (*Vespula* spp.) (rVes v 1), phospholipase A2 (*Apis mellifera*) (rApi m 1), and antigen 5 (*Vespula* spp.) (rVes v 5). All these rSSMA were free from CCDs. A sIgE values of ≥ 0.35 kUA/l were considered as positive. Allergic patients and healthy controls is shown in Table 2.

**Table 1:** Characteristics of patients allergic to *Hymenoptera* venom (test group) and healthy individuals (control group)

Sex	Allergic patients			Healthy controls		
	n	Median age	Mean age (range)	n	Median age	Mean age (range)
Male	12	31	31 (7–66)	26	64	53 (3–74)
Female	9	50	46 (9–67)	16	41	37 (5–74)
Total	21	36	37 (7–67)	42	54	46 (3–74)

**Table 2:** Percentage of positive values of specific IgE within the study groups

	Positive values of specific IgE determined to					
	WV	HBV	MUXF3 CCD	rVes v 1	rAp m 1	rVes v 5
Allergic patients	57.1%	100%	14.3%	23.8%	71.4%	33.3%
Healthy controls	4.8%	14.3	0%	2.4%	0%	0%

*WV* wasp venom, *HBV* honeybee venom, *MUXF3 CCD* cross-reactive carbohydrate determinant estimated to MUXF3 from bromelain, *rVes v 1* recombinant phospholipase A1 (*Vespula* spp.), *rAp m 1* recombinant phospholipase A2 (*Apis mellifera*), *rVes v 5* recombinant antigen 5 (*Vespula* spp.)

Blood samples obtained from study participants were incubated and centrifuged. Collected sera were stored in − 80 °C until analysis.

### Chemicals and reagents

Trifluoroacetic acid (TFA) and ammonium phosphate monobasic were supplied by Sigma Aldrich (St. Louis, MO, USA) and α-cyano-4-hydroxycinnamic acid (HCCA) was supplied by Bruker (Bremen, Germany). Acetonitrile (ACN), acetone, ethanol, and 2-propanol were supplied by J.T. Baker (Center Valley, PA, USA). The reagents were of analytical grade or better.

### Sample pretreatment

Before the MALDI-TOF MS (matrix-assisted laser desorption/ionization-time of flight mass spectrometry) analysis, purification, desalting, and concentration of biological material were performed. All serum samples were first diluted in 1:5 with 0.1% TFA in water and then loaded onto ZipTip C18 reverse phase chromatography pipette tips (Millipore, Bedford, MA, USA) according to manufacturer’s instruction. ACN and 0.1% TFA were used for prior tips conditioning. Bound peptides were washed with 0.1% TFA and eluted with 50% ACN in 0.1% TFA.

### MALDI-TOF MS analysis

After ZipTip purification and pre-concentration, 1 μl of each eluted sample fraction was mixed with ten microliters of daily prepared matrix solution (0.3 g/l HCCA in a 2:1 mixture of ethanol/acetone, *v/v*), then spotted onto AnchorChip Standard 800 µm target plate (Bruker Daltonics, Bremen, Germany) in triplicate and left in room temperature until crystallization. MS measurements were performed in a linear-positive mode with the use of MALDI-TOF/TOF UltrafleXtreme (Bruker Daltonics, Bremen, Germany) tandem mass spectrometer. To minimize systemic errors, blinded samples were analyzed in random order. The spectra were acquired from an average of 2000 laser shots per sample in the m/z range of 1000–10,000. It is reported, that MALDI-TOF MS provides optimal performance for this chosen m/z range, as for increasing peptide mass the resolution and detection efficiency progressively decrease. However, in the low m/z range (less than m/z of 1000) the higher background derived from ionized matrix molecules significantly impede detection of the peaks [10]. Analysis of three separate MALDI spots repetition was proceeded for each serum sample. External calibration was performed using a mixture of Protein Calibration Standard I and Peptide Calibration Standard (Bruker Daltonics, Bremen, Germany) (5:1, *v/v*). The average mass deviation was less than 100 ppm. For MALDI-TOF MS analysis the following parameters were used: ion source 1, 25.09 kV; ion source 2, 23.80 kV. Other applied settings were as follows: pulsed ion extraction, 260 ns, lens 6.40 kV, matrix suppression cut off m/z 700. To obtain average serum proteomic/peptidomic profiles of each study groups and for collection and processing of the spectra, FlexControl 3.4 (Bruker Daltonics, Bremen, Germany) software was applied. Inter-day and intra-day reproducibility of the spectra obtained after ZipTip depletion was evaluated in our previous study [11].

### Discriminative peaks identification

Identification of peptides with discriminatory power between allergic patients and healthy individuals is a crucial step for understanding the mechanism of pathological processes and gaining the knowledge about a disease progression [12]. Detection of thousands of proteomic compounds in different body fluids are possible through mass spectrometry techniques coupled with liquid chromatography [13]. Therefore, to increase the number of detectable peptides within the complex human serum sample, the MALDI-TOF MS/MS analysis was preceded with fractionation of the sample by nanoLC (nano-liquid chromatography) system. It resulted in proper baseline separation and precise precursor ion isolation. Moreover, the nanoLC separation step enables to overcome the ion supression in MALDI analysis of complex biological materials [14]. A serum sample was first pretreated with ZipTip C18 reverse phase chromatography pipette tips. The obtained undigested eluent (50% ACN, 0.1% TFA) was concentrated and subjected to nanoLC separation consisted of nanoflow HPLC (high performance liquid chromatography) system (EASY-nLC II, Bruker Daltonics, Bremen, Germany) and fraction collector (Proteineer-fc II, Bruker Daltonics, Bremen, Germany). The nanoLC set consisted of NS-MP-10 BioSphere C18 trap column for protein and peptide concentration (20 mm length, 100 µm inner diameter, pore size 120 Å, particle size 5 µm) (NanoSeparations, Nieuwkoop, the Netherlands) and Thermo Scientific Acclaim PepMap 100 column (150 mm length, 75 µm inner diameter, pore size 100 Å, particle size 3 µm) (Thermo Scientific, Sunnyvale, CA, USA) for separation. The linear gradient elution method was 2–50% of ACN in 96 min (mobile phase A: 0.1% TFA in water, mobile phase B: 0.1% TFA in ACN). The flow rate for separation was maintained at 300 nl/min, and the injected volume of the sample eluent was 2 µl. In total, 384 separated fractions were obtained. 80 nl of each fraction was mixed with 420 nl of matrix solution prepared of 36 µl of α-cyano-4-hydroxycinnamic acid saturated solution in 0.1% TFA and ACN (90:10, *v/v*), 784 µl of mixture of ACN and 0.1% TFA (95:5, *v/v*), 8 µl of 100 mM ammonium phosphate monobasic and 8 µl of 10% TFA and then automatically spotted onto AnchorChip 800 µm MALDI target plate using fraction collector. The nanoLC system was controlled with HyStar 3.2 software (Bruker Daltonics, Bremen, Germany). For the MS analysis, MALDI-TOF/TOF instrument (UltrafleXtreme, Bruker Daltonics, Bremen, Germany) working in a reflector mode in a mass range of m/z 700–3500 was applied. For the external calibration, Peptide Calibration Standard (Bruker Daltonics, Bremen, Germany) mixture was used. To establish a list of the precursor ions for the identification, WARP-LC (Bruker Daltonics, Bremen, Germany) software was used. Appointed m/z were analyzed with MS/MS mode. Settings for MS and MS/MS mode were: ion source 1, 7.50 kV; ion source 2, 6.75 kV; reflectron 1, 29.50 kV; reflectron 2, 14.00 kV; lens, 3.50 kV; lift 1, 19.00 kV; lift 2, 3.00 kV, pulsed ion extraction time, 80 ns. For the control of the MALDI-TOF MS instrument, spectra acquisition, processing and evaluation, FlexControl 3.4 (Bruker Daltonics, Bremen, Germany), FlexAnalysis 3.4 (Bruker Daltonics, Bremen, Germany) and BioTools 3.2 (Bruker Daltonics, Bremen, Germany) software was used. To analyze the results, SwissProt database and Mascot 2.4.1 search engine (Matrix Science, London, UK) were applied, searchers were taxonomically restricted to Homo sapiens. The following protein search parameters were used: precursor-ion mass tolerance ± 50 ppm; fragment-ion mass tolerance m/z ± 0.7; no enzyme; monoisotopic mass; peptide charge + 1.

### Data analysis

For the processing of the obtained MS spectra, comparison and statistical analysis, ClinProTools 3.0 (Bruker Daltonics, Bremen, Germany) chemometric software was used. Each serum sample was analyzed in triplicate using a mass spectrometer. Therefore, in order to classify corresponding repetitions as one biological replicate and average data, the function of spectra grouping was applied. Processing of spectra included recalibration using the prominent common m/z values, normalization to the total ion current (TIC), smoothing, the signal-to-noise ratio ≥ 5, baseline top hat subtraction (minimum baseline width: 10%), peak calculation and peak picking procedure. To improve the signal to noise ratio during peak picking operation, a total average spectrum was calculated. Spectra were processed and smoothed in the mass range of m/z 1000–10,000. Comparison between allergic patients and control group was evaluated with Wilcoxon test (statistical significance was considered when the p-value was ≤ 0.05). To get the most discriminative mathematical models which allow classifying test and control group, three algorithms were applied: genetic algorithm (GA), supervised neural network (SNN), and quick classifier (QC). The genetic algorithm relies on the process of natural selection and allows to determine the most discriminatory combinations of peaks basing on the idea of the evolution of the fittest individual. The supervised neural network algorithm chooses spectra characteristic to each of compared classes and based on them, classifies spectra to the corresponding group. Quick classifier algorithm calculates average areas of the peaks and uses p-values at a defined peak position for classification. Cross-validation, recognition capability and external validation parameters were calculated for each algorithm. The value of cross-validation is deemed to be a determinant of the reliability of the calculated model. It is a technique to evaluate the performance of a classifier. “Leave One Out” mode for calculating cross-validation was applied. This method was chosen regarding a number of samples.

Additionally, the receiver operating characteristic curve (for which area under the curve was calculated) was determined.

## Results

### Protein-peptide profiling

**Fig. 1:**
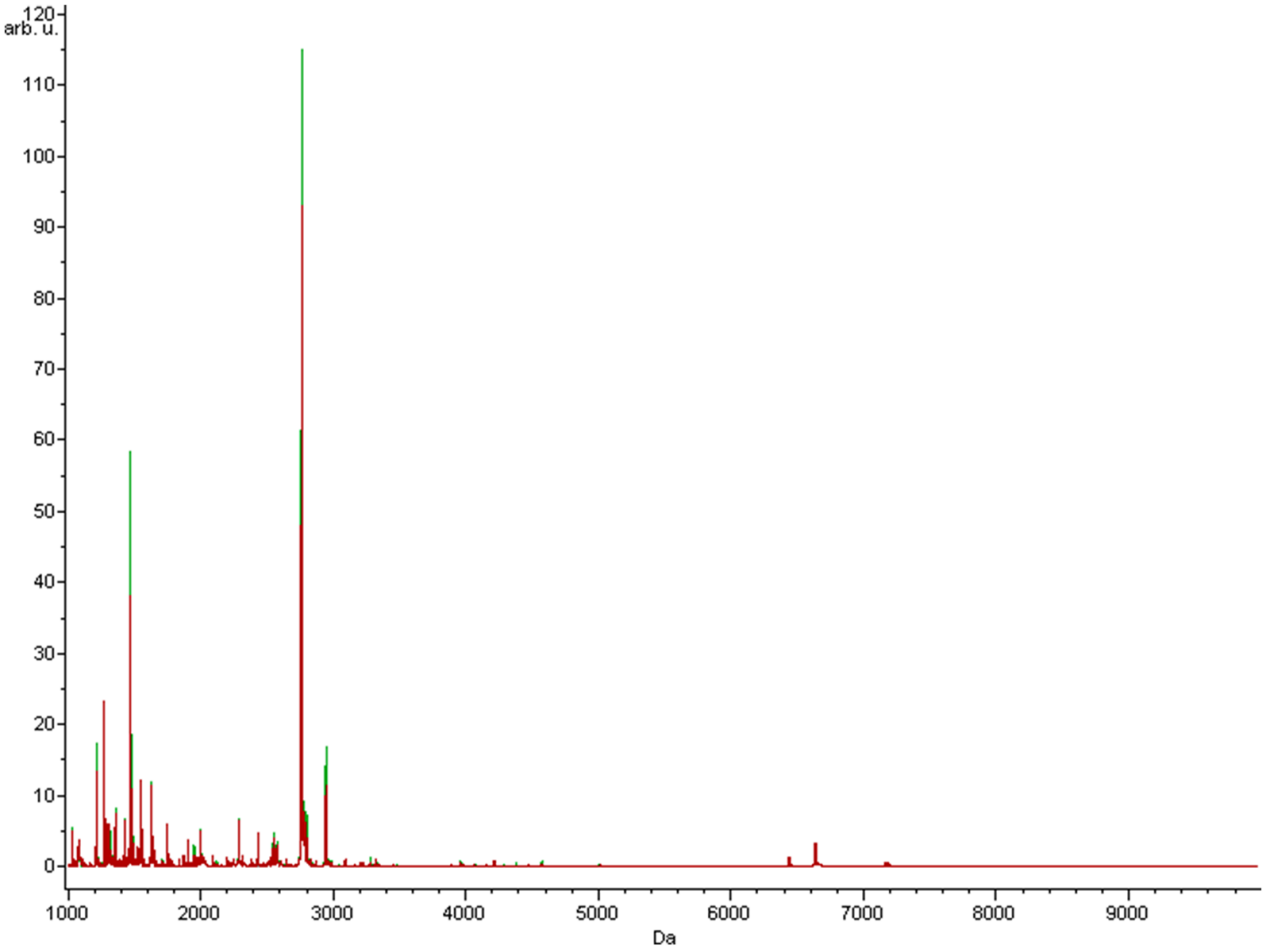
Average MALDI-TOF MS spectra of serum samples characteristic to study groups. Spectra of patients allergic to *Hymenoptera* venom (red) and healthy controls (green) are presented over the full scan range of m/z 1000–10,000

The average MALDI-TOF MS spectra characteristic to *Hymenoptera* venom allergic patients and healthy volunteers are presented in Fig. 1. These obtained data has been statistically calculated with three chemometric algorithms: genetic algorithm, supervised naural network, and quick classifier. All these algorithms vary in their methodology, hence peaks defined as differentiating for each of them are disparate (Table 3). Nevertheless, one peak of m/z 1627.76 is present both in genetic algorithm and supervised neural network. The highest value of average cross-validation from three repetitions (58.93%) was obtained using quick classifier. The highest recognition capability (87.86%) was appointed by the genetic algorithm. The greatest values of external validation were received for the quick classifier, the m/z of the discriminative peptide for this algorithm was 1066.17 (Table 4). The receiver operating characteristic (ROC) curve, for which the area under the curve (AUC) was calculated, was also determined. In the mass range of m/z 1000–10,000, the highest AUC value was obtained for a peptide of m/z 6431.3, classified as discriminative for model based on genetic algorithm.

**Table 3:** Peaks discriminating study groups generated by the applied algorithms

GA	SNN	QC
1466.67	1627.76	1066.17
1627.76	2795.18	
2012.02	3216.35	
2022.50		
2603.37		
3262.97		
3327.76		
5699.60		
6431.30		
6669.38		

*GA* genetic algorithm, *SNN* supervised neural network, *QC* quick classifier

**Table 4:** Values of chemometric parameters for the algorithms

	GA	SNN	QC
Cross validation (%)	52.68	45.36	58.93
Recognition capability (%)	87.86	50.30	66.19
Correct classified (%)			
Allergy	46.20	100.0	53.80
Control	75.90	34.50	69.00

*GA* genetic algorithm, *SNN* supervised neural network, *QC* quick classifier

### Discriminative peaks identification

The nanoLC-MALDI-TOF/TOF MS methodology proposed for this study allowed for the identification of four features differentiating between allergy and control groups. The analysis of precursor ion m/z 1466.67 resulted in the identification of fibrinogen alpha chain (FIBA_HUMAN) with significant Mascot hit based on the A.DSGEGDFLAEGGGVR.G peptide fragmentation sequence. Complement C4-A (CO4A_HUMAN) was another identified protein standing for the peak of m/z 1627.76 with the peptide sequence R.NGFKSHALQLNNRQ.I. Fragmentation of the precursor ion m/z 2012.02 resulted in peptide sequence P.GVLSSRQLGLPGPPDVPDHA.A with a high score in the Mascot database to inter-alpha-trypsin inhibitor heavy chain H4 (ITIH4_HUMAN). The fragmentation of signal m/z 2603.37 allowed us to identify the following sequence: R.AVPPNNSNAAEDDLPTVELQGVVPR.G. It gave a significant score in Mascot search to coagulation factor XIII chain A (F13A_HUMAN). The protein identification data is summarized in Table 5.

**Table 5:** List of identified proteins discriminative between *Hymenoptera* venom allergic patients and healthy controls

Precursor ion m/z	Protein ID	UniProtKB-ID	Peptide fragmentation sequence	Protein name
1466.67	P02671	FIBA_HUMAN	A.DSGEGDFLAEGGGVR.G	Fibrinogen alpha chain
1627.76	P0C0L4	CO4A_HUMAN	R.NGFKSHALQLNNRQ.I	Complement C4-A
2012.02	Q14624	ITIH4_HUMAN	P.GVLSSRQLGLPGPPDVP DHA.A	Inter-alpha-trypsin inhibitor heavy chain H4
2603.37	P00488	F13A_HUMAN	R.AVPPNNSNAAEDDLPT VELQGVVPR.G	Coagulation factor XIII A chain

Direct identification of discriminatory features is possible for peaks below m/z of 3500, as the high resolution of the analysis in the reflector mode is limited to low molecular weight peptides. Thus, the MS/MS analysis was conducted in the reflector mode in the mass range of m/z 700–3500. For this reason, differentiating peaks of m/z 5699.60, 6431.30 and 6669.38 could not be detected. Besides, discriminative peptides selected according to the analysis in the linear mode must be submitted to MS/MS analysis undigested. However, in non-tryptic peptides, fragmentation is often poor. That seems to be a problem, as information included in the databases refer mostly to the fragment ions derived from enzymatic digestion, with lysine and arginine residues in N-terminal and C-terminal regions. Moreover, the presence of neighboring peaks may impede unambiguous identification. Hence, the identification of m/z 2022.50, 3262.97 and 3327.76 requires further analysis.

## Discussion

Proper diagnosis and management of *Hymenoptera* venom sensitization require a basic knowledge of the molecular mechanism of allergy development. Thus, in this study, we aimed to assess the alterations in low molecular peptide and protein composition in blood after *Hymenoptera* venom sensitization. Because exposure to *Hymenoptera* venom in allergic subjects may result in allergic inflammation entailing changes in structure and function of the affected cells [6, 15], we described the identified discriminatory features regarding their contribution to the inflammatory conditions.

**Fig. 2:**
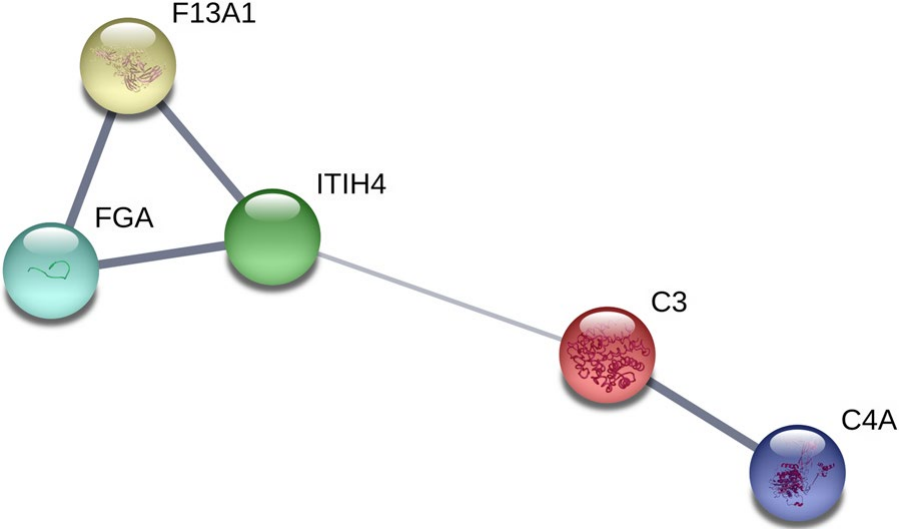
Interactions between proteins differentiating patients allergic to *Hymenoptera* venom and healthy individuals. *F13A1* coagulation factor XIII, *FGA* fibrinogen alpha chain, *ITIH4* inter-alpha-trypsin inhibitor heavy chain H4, *C3* complement C3, *C4A* complement C4-A (Source: https://string-db.org)

The development of localized and systemic allergic reactions following *Hymenoptera* sting is mostly related to allergen-specific immunoglobulin E (IgE) antibodies. Consequently, the inflammation mediators are released to neutralize toxins and restore homeostasis [16–18]. In the presented study, a proposed methodology allowed for the identification of four inflammation factors involved in the development of *Hymenoptera* venom allergy and pathological processes following the sting. They were: fibrinogen alpha chain, complement C4-A, inter-alpha-trypsin inhibitor heavy chain H4 and coagulation factor XIII chain A. The interactions between identified features, are shown in Fig. 2.

Fibrinogen and coagulation factor XIII participate in blood clotting, mediating aggregation. It is reported, that the balance between coagulation and inflammation is crucial to obtain the protection from various environmental, pathological or mechanical factors. These two pathways are initiated by the same types of event and factors. Moreover, they are observed to occur in the same types of tissues, organs, and pathologies [19]. During inflammation, fibrinogen is enzymatically converted to fibrin, which is stabilized by activated coagulation factor XIII [20–22]. These factors, along with other acute-phase proteins, restrain the spreading of inflammation and eliminate its consequences, as these proteins, participating in clot formation, play a role in platelets and toxins removal [18]. Because of its antioxidant properties, fibrinogen may also protect from oxidative stress arising from inflammation [23]. According to the literature, concentrations of fibrinogen are reported to be differentiated in such allergic diseases as allergic asthma [24] and allergic rhinitis [25]. The precursor ion of m/z 1466.67 (in this presented research identified as fibrinogen alpha chain) was also classified as discriminative in our previous study, comparing protein-peptide patterns of stung and non-stung beekeepers [8]. That confirms the role of the fibrinogen in the allergic inflammatory response. The slight differences between m/z values presented in this study and previous research are associated with methods of spectra normalization and deviation of calibration.

Factors formed in the process of coagulation are involved in activation of the complement system and producing of kinin [18]. Complement activation is recognized as a central event of inflammation. Molecules resulting from proteolytic cleavage of complement proteins act as chemoattractants, antimicrobials, opsonizes and proinflammatory mediators [26]. Thus, the complement system is crucial for cellular integrity and homeostasis. Moreover, it plays an important role in adaptive immune response [27]. In the presented study, we identified complement C4-A as a feature differentiating allergic individuals and control group. Fragment of this protein was identified for the peak of m/z 1627.76 classified as discriminative in both genetic algorithms and supervised neural network. Human complement C4-A is a non-enzymatic component of the C3 convertase [28]. Thus, being a part of the classical complement pathway, it is a mediator of the local inflammatory process. Complement C4-A causes contraction of smooth muscle, release histamine and increases vascular permeability. It also activates immunological pathways, playing a role in immune response [29]. The importance of the complement C4-A in the development of inflammatory response was also confirmed in our previous study [8]. The peak of m/z 1627.76, standing for complement C4-A, was classified as differentiating between stung and non-stung individuals.

It is reported, that excessive production of the complement C4-A may result in the overreaction of the complement pathway, exacerbating the inflammatory response. Therefore, complement inhibitors seem to be essential to avoid detrimental excess activation consequences. The protein with the potential to bind complement and attenuate its activation is inter-alpha-trypsin inhibitor heavy chain H4. It may inhibit both classical and alternative complement pathway [26]. In this study, ITIH4 was the last identified feature differentiating the studied groups. It is an acute-phase plasma glycoprotein belonging to heavy-chain inter-alpha-trypsin inhibitor family [30]. Although the exact function of the ITIH4 is not known, it appears in human as a result of inflammation, stress or trauma [31].

## Conclusions

Analytical and bioinformatics strategy proposed for this study allowed for the determination of the mathematical models distinguishing pathological (*Hymenoptera* venom allergy) and normal state. The application of MALDI-TOF MS technique enabled protein-peptide profiling and identification of four protein features responsible for an inflammatory response in venom allergic patients. They were: fibrinogen alpha chain, coagulation factor XIII chain A, complement C4-A, and inter-alpha-trypsin inhibitor heavy chain H4. So far, any reports characterizing the proteomic/peptidomic origin of *Hymenoptera* venom allergy have been published. Extending the knowledge of the *Hymenoptera* venom sensitization will undoubtedly contribute to the development of novel, sensitive and specific methods for quick and unambiguous allergy diagnosis. Understanding the basis of the allergy at the proteomic level will support the improvement of preventive and therapeutic measures. Due to the risk of life-threating anaphylactic reactions following exposure to *Hymenoptera* venom, implementation of advanced prognostic, diagnostic and treatment strategies is urgently required. This study is the first step towards the comprehensive management of *Hymenoptera* venom allergy, which will result in the enhancement of human well-being.

## Data Availability

The datasets supporting the conclusions of this article are included within the article. More datasets of the current study are available from the corresponding author on reasonable request.

## Abbreviations

MALDI-TOF MS: matrix-assisted laser desorption/ionization-time of flight mass spectrometry
VIT: venom immunotherapy
sIgE: venom specific immunoglobulin E
CCD: cross-reactive carbohydrate determinant
MUXF3: neo-glycoprotein fucosylated/xylosylated *N*-glycans
rSSMA: species-specific recombinant major allergens
rVes v 1: phospholipase A1 (*Vespula spp*.)
rApi m 1: phospholipase A2 (*Apis mellifera*)
rVes v 5: antigen 5 (*Vespula spp*.)
TFA: trifluoroacetic acid
HCCA: α-cyano-4-hydroxycinnamic acid
ACN: acetonitrile
nanoLC: nano-liquid chromatography
HPLC: high performance liquid chromatography
TIC: total ion current
GA: genetic algorithm
SNN: supervised neural network
QC: quick classifier
ROC: receiver operating characteristic
AUC: area under the curve
FIBA_HUMAN: fibrinogen alpha chain
CO4A_HUMAN: complement C4-A
ITIH4_HUMAN: inter-alpha-trypsin inhibitor heavy chain H4
F13A_HUMAN: coagulation factor XIII chain A
